# Radioisotopes Demonstrate the Contrasting Bioaccumulation Capacities of Heavy Metals in Embryonic Stages of Cephalopod Species

**DOI:** 10.1371/journal.pone.0027653

**Published:** 2011-11-23

**Authors:** Thomas Lacoue-Labarthe, Roger Villanueva, Claude Rouleau, François Oberhänsli, Jean-Louis Teyssié, Ross Jeffree, Paco Bustamante

**Affiliations:** 1 International Atomic Energy Agency – Environment Laboratories, Monaco; 2 Littoral Environnement et Sociétés (LIENSs), UMR 6250 CNRS – Université de La Rochelle, La Rochelle, France; 3 Institut de Ciències del Mar, CSIC, Passeig Maritim de la Barceloneta 37–49, Barcelona, Spain; 4 Institut Maurice-Lamontagne, Mont-Joli, Canada; 5 Faculty of Science, University of Technology Sydney, Sydney, Australia; Biodiversity Insitute of Ontario - University of Guelph, Canada

## Abstract

Cephalopods play a key role in many marine trophic food webs and also constitute alternative fishery resources in the context of the ongoing decline in finfish stocks. Most coastal cephalopod species of commercial importance migrate into shallow waters during the breeding season to lay their eggs, and are consequently subjected to coastal contamination. Eggs of common cuttlefish *Sepia officinalis*, European squid *Loligo vulgaris*, common octopus *Octopus vulgaris* and the sepiolid *Rossia macrosoma* were exposed during embryonic development to dissolved ^110m^Ag, ^109^Cd, ^60^Co, ^54^Mn and ^65^Zn in order to determine their metal accumulation efficiencies and distribution among different egg compartments. Cuttlefish eggs, in which hard shells enclose the embryos, showed the lowest concentration factor (CF) values despite a longer duration of exposure. In contrast, octopus eggs, which are only protected by the chorionic membrane, accumulated the most metal. Uptake appears to be linked to the selective retention properties of the egg envelopes with respect to each element. The study also demonstrated that the octopus embryo accumulated ^110m^Ag directly from the dissolved phase and also indirectly through assimilation of the contaminated yolk. These results raise questions regarding the potential contrasting vulnerability of early life stages of cephalopods to the metallic contamination of coastal waters.

## Introduction

Cephalopods in European waters have attracted increasing attention from marine biologists and fishery scientists [1]. Indeed, this group is of high ecological significance for the understanding of the food webs in marine ecosystems. This reflects their central position as they are as much a predator of fish and invertebrates as they are prey for numerous fish, seabirds and marine mammals [2], [3]. Secondly, cephalopod fisheries have increased significantly over the last six decades, with landings nowadays reaching 120 000 tons per year, i.e., 4-fold the landings made in the 1950 s [4], and they therefore constitute alternative resources in the context of the ongoing decline in finfish stocks. The European squid *Loligo vulgaris*, the common octopus *Octopus vulgaris* and the common cuttlefish *Sepia officinalis* are the main targets of these fishery activities in Europe [1]. For these reasons, an increasing number of studies has focused on their biology, ecology and their responses to the environmental factors that drive their population dynamics.

These coastal cephalopods undergo extensive (e.g., *S. officinalis*) or limited migration (e.g., *O. vulgaris*) in the breeding season to reach shallow waters, where they lay their eggs on benthic substrates. The eggs therefore develop in an environment that is exposed to more intense anthropogenic activities than adult environments, and to the release of various contaminants including metals. Cephalopod embryos are thus exposed to dissolved trace elements during development, which represents 10–15% of the cephalopod life span [5]. Previous studies of *S. officinalis* demonstrated that its embryos, which are encapsulated in an eggshell composed of nidamental-, oviducal-originated layers and a chorion, are partially protected against metal penetration [6]–[8]. Interestingly, these shielding properties varied with respect to the particular trace element, resulting in, for instance, high Ag-accumulation in the embryonic tissues but total protection against Pb-penetration [9]–[10]. This contrasting metal-dependent permeability of the eggshell can thus limit the exposure of the embryo to the potential toxicological effects of particular contaminants during development, but not others [11].

Within the cephalopods, egg encapsulation varies in terms of size, architecture and structure [12]. Briefly, common cuttlefish and sepiolids lay individual medium-sized encapsulated eggs that are covered by gelatinous envelopes stained with ink or by a jelly covered by a rigid capsule, respectively [12]. European squid lay several small-sized eggs that are united by a common oviducal and nidamental jelly sheet, whereas the common octopus produces small eggs that are devoid of an envelope other than the chorion [13]. In this context, this study aimed to determine the potential inter-specific differences in metal bioaccumulation in the embryos and hatchlings of four species (three of which are of high economic interest) that differ appreciably in their eggshell structure. By exposing the eggs to dissolved ^110m^Ag, ^109^Cd, ^57^Co, ^54^Mn and ^65^Zn throughout development, we determined the concentration and distribution of radiotracers in the eggshell envelopes, vitellus and embryo to help explain the bioaccumulation efficiencies observed in hatchlings of four species, *R. macrosoma*, *S. officinalis*, *L. vulgaris*, and *O. vulgaris*, representing the three main Orders of the Class Cephalopoda.

## Materials and Methods

Adult cuttlefish and octopus, collected by net fishing off Monaco in March 2006 and June 2008, respectively, were maintained in 300-l open-circuit tanks at the IAEA-EL premises and fed daily with crabs and frozen fish. After spawning, cuttlefish eggs (n = 20) were collected and immediately separated to optimise their oxygenation. Octopus egg masses (six strings of ∼200 eggs each) were kept with the female for brooding during the first week to optimise embryo survival before their collection for experiments. Freshly laid eggs of squid (n = six strands of ∼40 eggs each) and sepiolids (n = 12 eggs) were collected in May 2008 and March 2009, respectively, off Monaco at a depth of 20–300 m, and placed immediately under experimental conditions.

The eggs were placed in four 20-l glass aquaria containing filtered (0.45 µm) and UV-sterilized seawater (constantly aerated closed circuit; salinity 37 p.s.u.; light/dark cycle: 12 h/12 h) at 16°C, or 19°C for the octopus eggs. They were then exposed to dissolved radiotracers ^110m^Ag, ^109^Cd, ^57^Co (Amersham, UK) and ^54^Mn and ^65^Zn (Isotope Product Laboratory, USA) throughout development (except octopus eggs, which were exposed one week after spawning), according to the experimental procedure described by Lacoue-Labarthe et al. [14]. Due to the sensitivity of the radiotracer tool, the low levels of radioactivity used in this work allowed us to monitor trace element accumulation behaviour under environmentally realistic levels of metal exposure [15]. The concentrations of radiotracers in seawater, exposure durations and concentrations of added metals are reported in Table 1.

**Table 1:** Exposure duration expressed in days (d) and radiotracer activities in seawater (mean ± SD) during experimental exposure.

	*R. macrosoma*	*S. officinalis*	*L. vulgaris*	*O. vulgaris*
Exposure duration (d)	82	44	23	28
^110m^Ag (kBq.l^−1^)	0.45±0.33	0.66±0.37	0.55±0.37	0.54±0.37
^109^Cd (kBq.l^−1^)	1.41±0.58	1.56±0.77	1.71±0.37	0.94±0.47
^57^Co (kBq.l^−1^)	1.05±0.38	1.02±0.44	0.73±0.13	0.80±0.31
^54^Mn (kBq.l^−1^)	1.10±0.46	1.07±0.40	0.75±0.11	0.93±0.14
^65^Zn (kBq.l^−1^)	0.80±0.46	0.93±0.45	0.78±0.12	0.64±0.31
Ag (ng.l^−1^)	108	1.3	86	86
Cd (ng.l^−1^)	16	0.1	na	na
Co (ng.l^−1^)	2.10^−3^	1.2	170	170
Mn (ng.l^−1^)	7.10^−2^	25.10^−2^	na	na
Zn (ng.l^−1^)	1.2	65.10^−3^	64	64

na: non available data.

The eggs were sampled at intermediate developmental stages [16] (Table 2) (n = 6 and 4 eggs for sepiolids and cuttlefish, respectively; n = 9 pools of 10–20 eggs from three strands of squid and five pools of 20 eggs for octopus, to obtain sufficient biomass to allow the detection of significant levels of radiotracers) and dissected to determine the distribution of the radiotracers among the different egg compartments, *i.e.* the different eggshell layers, the embryo, the vitellus and the perivitelline fluid. At hatching, six and four sepiolid and cuttlefish hatchlings, respectively, and nine pools of 10–20 individuals and five pools of 25 individuals of squid and octopus hatchlings, respectively, were weighed and counted.

**Table 2:** Concentration factors (mean ± SD) of ^110m^Ag, ^109^Cd, ^57^Co, ^54^Mn, and ^65^Zn in the hatchlings (wet weight; mg) and in the compartments of eggs sampled at intermediate embryonic developmental stages (according to Naef [16]) of *Rossia macrosoma*, *Sepia officinalis*, *Loligo vulgaris* and *Octopus vulgaris* exposed to dissolved radiotracers in seawater during embryonic development (days; d).

Species	*R. macrosoma*	*S. officinalis*	*L. vulgaris*	*O. vulgaris*
**Hatchlings**				
Wet weight	121.6±3.9	137.1±11.3	3.9±0.9	1.3±0.1
^110m^Ag	398±44	2025±176	5504±2781	20760±3785
^109^Cd	41±19	83±17	150±75	1111±366
^57^Co	6±4	161±13	54±18	221±73
^54^Mn	29±3	92±4	66±19	120±23
^65^Zn	62±65	656±53	1515±348	2669±183
**Egg compartments**				
Developmental stages	XVII	XVIII	XVII	XVI
**^110m^Ag CF**				
Whole eggshell	5274±788	1846±223	595±95	2147±181
Nidamental	6907±781	2180±245	894±241	-
Oviducal	<dl	310±154	307±262	-
Chorion	<dl		114±95	2147±181
Embryo	<dl	5253±891	997±1245	1525±284
Vitellus	<dl	1242±477	398±393	6953±1067
PVF	<dl	27.8±9.1	8.3±7.0	na
**^109^Cd CF**				
Whole eggshell	3158±635	590±238	74.4±7.6	<300
Nidamental	12295±1990	663±274	40±8	-
Oviducal	134±28	237±73	73±24	-
Chorion	<dl		169±33	<300
Embryo	<dl	<dl	51.4±33.3	595±186
Vitellus	<dl	<dl	136±104	433±165
PVF	<dl	<dl	22.2±8.0	na
**^57^Co CF**				
Whole eggshell	1249±308	1522±298	109±27	1151±177
Nidamental	4009±1055	1550±311	33±10	-
Oviducal	28±22	1151±385	75±44	-
Chorion	<dl		332±109	1151±177
Embryo	<dl	<dl	25.0±9.3	48.4±89.0
Vitellus	<dl	<dl	21.0±10.9	<10
PVF	<dl	<dl	24.3±9.2	na
**^54^Mn CF**				
Whole eggshell	217±9	174±45	137±40	489±160
Nidamental	646±65	200±54	159±53	-
Oviducal	56±12	52±11	121±26	-
Chorion	133±77		75±16	489±160
Embryo	27.9±1.7	33.6±4.1	35.2±19.8	<20
Vitellus	2.5±1.3	5.23±2.71	26.5±10.7	<5
PVF	5.4±2.7	3.9±0.6	3.4±4.8	na
**^65^Zn CF**				
Whole eggshell	5531±997	1359±169	146±25	2929±370
Nidamental	12474±2116	1490±678	182±31	-
Oviducal	42±36	678±205	125±44	-
Chorion	<dl		66±14	2929±370
Embryo	<dl	335±185	1136±428	164±30
Vitellus	<dl	194±133	1032±385	99±26
PVF	<dl	1.2±0.4	10.0±1.5	na

na: not analysed; dl: detection limit; PVF, perivitelline fluid.

Radioanalyses were carried out using a high-resolution γ-spectrometry system consisting of four coaxial Germanium (N- or P-type) detectors (EGNC 33-195-R, Canberra® and Eurysis®) connected to a multi-channel analyser and a computer equipped with spectra analysis software (Interwinner® 6). Radioactivities were determined by comparison with standards of known activity and of appropriate geometry. Measurements were corrected for counting efficiency and physical radioactive decay. The counting time was adjusted to obtain a propagated counting error of less than 5% [17].

The accumulation of each radiotracer was expressed as a concentration factor (CF), which is the ratio between radiotracer activity in the egg or egg compartment (Bq g^−1^) and the time-integrated activity in seawater (Bq g^−1^). The results are expressed as mean ± SD.

Whole-body autoradiography was used to reveal the distribution of ^110m^Ag in octopus eggs exposed to dissolved ^110m^Ag for 1 week. Eggs were embedded in a 2.5% carboxymethylcellulose gel and flash-frozen in a slurry of dry ice in hexane. From these egg pools, 20-µm thick sections were cut with a specially designed cryomicrotome (Leica CM3600), freeze-dried, and placed on phosphor screens (Perkin-Elmer) for 4 to 7 d. After exposure, the screens were scanned with a Cyclone Phosphor Imager (Perkin-Elmer) and ^110m^Ag activity in the egg compartments was quantified as previously described [18].

A Kruskal-Wallis test was applied to determine the differences between the CFs calculated for the different egg compartments. All experiments complied with the ethical and welfare considerations relating to the use of cephalopods as experimental animals [19].

## Results and Discussion

Many studies have shown that cephalopods have the capacity to accumulate trace elements at high levels in their tissues [20]–[28]. For this reason, the health concerns related to the consumption of cephalopod flesh have been evaluated for different commercial species from different locations [29]–[33]. In cephalopods, the effects of metals range from direct effects during embryogenesis to various toxic effects on the early juveniles [11], [34]–[36]. Nonetheless, both maternal transferred and dissolved metals can have an impact during the embryo's development, and may be particularly critical for the success of population recruitment in cephalopods.

This study revealed that metal accumulation (considering all radioisotopes) in embryos varied between the four considered species, such that CF_octopus_>CF_squid_≥CF_cuttlefish_>CF_sepiolid_ (Table 2). Although the maternal transfer of several elements has previously been demonstrated through the vitellus [37], our results are consistent with the natural metal concentrations recorded in hatchlings from the field [38]. This suggests that metal concentrations in hatchlings reflect the environmental conditions in which the eggs develop and depend on egg morphology. Once the eggs have been laid, the observed inter-specific differences in bioaccumulation therefore result from contrasting waterborne metal uptake that is likely to be due to the type of encapsulation of the embryos, which is specific to the different species. The CF values recorded in embryos at intermediate stages of development (Table 2) suggest differences in the permeabilities of the eggshells towards metals. Indeed, all radiotracers accumulated in the embryonic tissues in squid and octopus whereas only ^54^Mn and ^110m^Ag, and ^54^Mn and ^65^Zn, respectively, were detected at significant levels in the internal compartment of sepiolid and cuttlefish eggs.

The selective permeability of the cuttlefish eggshell towards trace elements has previously been interpreted as being linked to the expansion of the chorionic membrane, resulting in egg swelling [9], [10], [14], which induces the shrinkage and stretching of the envelopes due to the notable increase in egg volume [12]. Nevertheless, the composition of the eggshell, which is rich in carboxyl- and sulphydryl-groups [13], might explain why most metals were associated with this compartment (>90% for ^109^Cd, ^57^Co, ^54^Mn, ^65^Zn and >64% for ^110m^Ag; results not shown), therefore hindering metal penetration into the internal compartments. More precisely, ^110m^Ag, ^109^Cd, ^54^Mn and ^65^Zn were more highly concentrated in the nidamental layers than in the pooled oviducal envelopes and chorion, but ^57^Co was found at similar concentrations in these two compartments (P*_Kruskall-Wallis_*>0.05; Table 2), suggesting that different components of the eggshell provide different levels of shielding according to the metal. The contrasting affinities of metals for the different eggshell parts were more obvious in squid eggs. Thus, the chorion may act as a barrier against ^109^Cd and ^57^Co penetration as it exhibited CF values 4 and 10-fold higher (P*_Kruskall-Wallis_*<0.01; Table 2) than the nidamental layers and retained 41 and 60% of the total amount of metal accumulated, respectively. In contrast, ^110m^Ag, ^54^Mn and ^65^Zn were mainly bound to the nidamental- and oviducal-originated layers (P*_Kruskall-Wallis_*<0.01). These results highlight the fact that the diffusion capacities of the different metals through the egg envelopes differ with respect to the element considered and to the eggshell composition and/or structure. Thus, the soft and gelatinous eggshell of squid appears more permeable than this of cuttlefish leading to higher accumulation of heavy metals in the embryo during development.

With regard to the “well-protected” egg of sepiolids, more than 99% (results not shown) of ^110m^Ag, ^109^Cd, ^57^Co and ^65^Zn was bound to the outer “nidamental” envelope, with the highest CF values recorded in this compartment among the four species investigated (Table 2). In contrast to squid and cuttlefish eggs, the outer coating in sepiolids is a rigid sphere. Therefore, its volume does not change due to the egg swelling that occurs during development. Moreover, its structure is characterized by several layers, each covered by an electron microscopic “dense” membrane [39] that might favour the retention of metal ions. Such strong shielding properties of the eggshell probably explain the low metal contamination found in the hatchlings despite long-term exposure (i.e., 82 d) to radioisotopes. It is therefore likely that radiotracer uptake occurs rapidly during the last days of embryonic life when the eggcase starts to break before hatching. However, ^54^Mn was detected in the internal jelly and chorion, suggesting that this essential element can pass selectively through the outer capsule before accumulating in the embryo.

**Figure 1:**
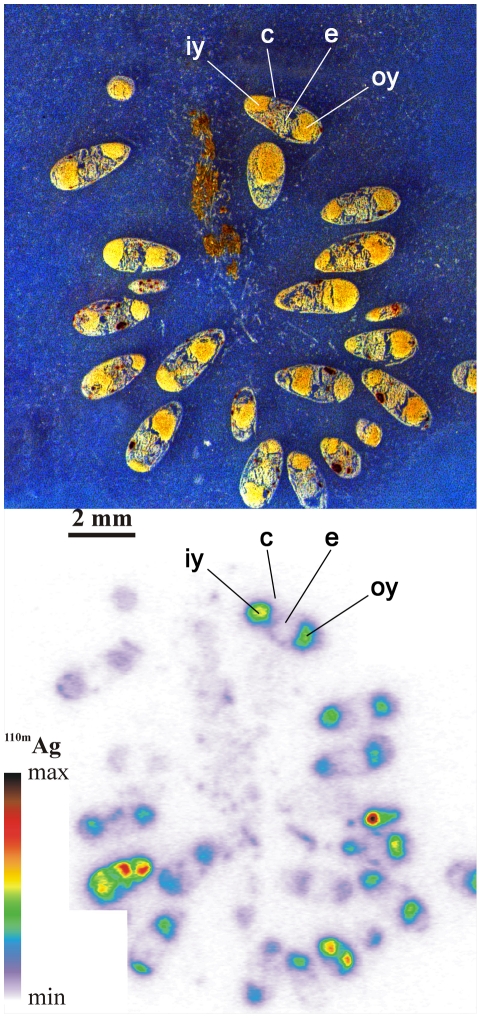
Tissue section (top) and autoradiogram (bottom) of *Octopus vulgaris* eggs at stage XVI [**16**] after exposure to dissolved ^110m^Ag for 1 week; c: chorion, e: embryo, iy: inner yolk sac, oy: outer yolk sac.

Octopus eggs lack an eggshell and the eggs are only protected by a chorionic membrane, which retained 88, 84 and 72% of the ^57^Co, ^54^Mn and ^65^Zn, respectively (results not shown). However, the retention capacity of the chorion for ^110m^Ag and ^109^Cd was low (i.e., 4 and <7%, respectively). Consequently, both metals became highly concentrated in the internal compartments (Table 2), resulting in at least 4- and 7-fold higher CF values in the octopus hatchlings than in the other species (Table 1). Our results are consistent with the 5-fold higher Ag concentrations recorded in hatchlings of *O. vulgaris* compared to those of *S. officinalis* and *L. vulgaris* [38]. In cuttlefish, it is noteworthy that the embryo accumulated more of the metals that penetrated through the eggshell than the other compartments (*i.e.* CF_emb_>CF_yolk_>CF_PVF_; P*_Kruskall-Wallis_*<0.05; Table 2). In contrast, no significant differences between the embryo and the yolk were observed in the CF values of ^65^Zn in cuttlefish, ^57^Co, ^54^Mn, ^65^Zn in squid and ^109^Cd in octopus (P*_Kruskall-Wallis_*>0.05; Table 2). More surprising was that the highest CF values of ^109^Cd and ^110m^Ag were observed in the yolk of squid and octopus, respectively, compared to the other compartments (P*_Kruskall-Wallis_*<0.01; Table 2). The autoradiogram of octopus eggs exposed to dissolved ^110m^Ag revealed that metal distribution was closely related to the position of the outer and inner yolk sacs (Figure 1). This clearly indicates that Ag is efficiently incorporated in the vitelline reserves. Thus, the octopus embryo accumulates waterborne Ag both directly from seawater and from the yolk, which absorbs the metal. This would explain the high CF values recorded in octopus paralarvae at hatching (CF_Ag_ of 20760±3785; Table 2). In this context, the embryos of octopus appeared to be the most highly exposed to environmental contamination by metals, and especially by Ag.

In conclusion, this work shows the strongly contrasting efficiencies of metal uptake by the four studied species, which appears to be correlated with the different embryo encapsulation strategies observed in these cephalopods [12]. The lowest CF values were found in the sepiolid and cuttlefish embryos (despite their longer exposure to radiotracers in this study; see Table 2), both of which are protected by hard shells during their longer embryonic life, unlike those of squid, which are embedded in soft envelopes, or those of octopus that lack any protective eggshell [12]. The large eggs of *R. macrosoma* and *S. officinalis* produce large, benthic hatchlings that weigh 30 to 100 times more than the small, planktonic and active swimming hatchlings of *L. vulgaris* and *O. vulgaris* [5], [40] (Table 2). The different mode of life of these early stages suggests the possibility of a contrasting metabolism between the embryos that could modulate the rate of uptake of metals [41]. In fact, metals were incorporated in inverse relation to body size (i.e. lower for the large-sized *S. officinalis* and higher for the small-sized *O. vulgaris*). It can thus be tentatively hypothesized that the surface–volume relationship may influence metal absorption [42], [43]. Finally, these results demonstrate different strategies regarding metal requirements and/or protection with respect to the essential and non-essential elements [37].
